# Integration of hereditary thrombophilia data and polygenic risk scores into prognostic models improves differential diagnosis of CTEPH

**DOI:** 10.3389/fcvm.2026.1887880

**Published:** 2026-10-05

**Authors:** Yaroslav Kubkin, Dmitry Nikulin, Galina Okhrimenko, Larisa Minushkina, Elizaveta Dyachkova, Anna Lyzhenkova, Anna Danilova, Elena Dankovtseva, Irina Borovikova, Vladimir Zamyatin, Alexander Averyanov, Maria Poptsova, Dmitry Zateyshchikov

**Affiliations:** 1 Centre for Biomedical Research and Technologies, Institute of Artificial Intelligence and Digital Sciences, HSE University, Moscow, Russia; 2 City Clinical Hospital No 29 by N.E. Bauman, Moscow, Russia; 3 Faculty of Physics, Lomonosov Moscow State University, Moscow, Russia; 4 Faculty of Bioengineering and Bioinformatics, Lomonosov Moscow State University, Moscow, Russia

**Keywords:** chronic thromboembolic pulmonary hypertension, deep learning, hereditary thrombophilia, meta-analysis, polygenic risk score, prognostic systems, pulmonary arterial hypertension

## Abstract

**Background:**

Differential diagnosis between pulmonary arterial hypertension (PAH) and chronic thromboembolic pulmonary hypertension (CTEPH) is crucial for treatment selection, particularly regarding surgical intervention. The prevalence of hereditary thrombophilias in Russian CTEPH patients and the utility of genetic data in differential diagnosis remain understudied.

**Objectives:**

To evaluate the prevalence of inherited thrombophilia among patients with CTEPH and PAH, to benchmark the results against published data from other cohorts, and to assess the potential utility of integrating genetic findings and polygenic risk score (PRS) parameters with clinical data for differentiating CTEPH from PAH.

**Methods:**

The study included 390 patients (252 PAH, 138 CTEPH). Whole-genome sequencing was performed to analyze classical thrombophilia genes (*PROC, PROS*1*, SERPINC*1*, F*2*, F*5*, ADAMTS*13*, HRG, JAK*2*, PIGA, THBD, F*9*, FGA, FGB, FGG, CALR*) and calculate VTE polygenic risk scores. A meta-analysis of 15 studies on hereditary thrombophilia prevalence in CTEPH was conducted. AutoML algorithm was used to construct diagnostic models.

**Results:**

Rare variants in thrombosis-associated genes were significantly more prevalent in patients with CTEPH than in those with PAH (12.3% vs. 1.2%, *p* < 0.001). Similarly, carriers of the common F5 and F2 thrombophilia variants were more frequent in the CTEPH group (15.9% vs. 6.3%, *p* = 0.002). Meta-analysis yielded pooled prevalence estimates in CTEPH of 3.0% for antithrombin deficiency, 3.6% for protein S deficiency, 4.7% for protein C deficiency, 7.9% for F5 Leiden, and 5.6% for F2 G20210A; these estimates were consistent with those reported in other populations. The clinical model yielded an ROC AUC of 0.883. The addition of PGS004854 and rare variants in thrombosis-associated genes increased the AUC to 0.951, representing an absolute increase of 0.068 (7.7%; *p* = 0.026). In the multivariable model, PGS004854 was the strongest positive genetic predictor of CTEPH (*β* = 1.869, *p* < 0.001).

**Conclusions:**

The genetic profile of CTEPH patients differs significantly from PAH, with a higher frequency of hereditary thrombotic risk factors. Integrating clinical data with polygenic risk scores and thrombophilia variants improves differential diagnosis accuracy and may optimize the clinical workup.

## 1 Introduction

Pulmonary arterial hypertension (PAH) and chronic thromboembolic pulmonary hypertension (CTEPH) represent precapillary forms of pulmonary hypertension for which PAH-specific therapies have been established (1). For CTEPH, surgical intervention is also available and significantly improves survival outcomes. Early detection of thrombotic lesions in the pulmonary arteries is essential for timely patient referral to expert centers, evaluation of surgical eligibility, and selection of appropriate anticoagulant therapy. Advances in diagnostics have led to PAH increasingly manifesting at an older age, often in patients with multiple comorbidities (2). While a history of symptomatic venous thromboembolism is common in patients with right heart failure of various etiologies, conversely, approximately 20% of CTEPH patients have no documented history of such episodes (3).

Hereditary disorders of hemostasis are recognized risk factors for CTEPH; however, they are not considered the primary drivers of the transformation from acute pulmonary embolism to chronic disease. To date, data regarding the prevalence of such hereditary defects among Russian CTEPH patients remain unavailable. Furthermore, all attempts to identify a specific genetic background for this condition—similar to that established for idiopathic PAH—have been unsuccessful. Consequently, current clinical recommendations for genetic screening are restricted to patients with PAH (4). Recently, however, there has been a growing interest in more complex genomic approaches, specifically the application of polygenic risk scores (PRS), to better characterize the genetic architecture of thromboembolic diseases.

The present study aimed to evaluate the prevalence of inherited thrombophilia among patients with CTEPH and PAH, to benchmark the results against published data from other cohorts, and to assess the potential utility of integrating genetic findings and polygenic risk score parameters with clinical data for differentiating CTEPH from PAH.

## 2 Materials and methods

### 2.1 Patient characteristics and study protocol

Consecutive patients with pulmonary hypertension [Group 1: PAH and Group 4: Pulmonary hypertension associated with pulmonary artery obstructions according to the current ESC/ERS clinical guidelines (5)] admitted to the Moscow Expert Center between January 1, 2014, and December 31, 2025, having signed an informed consent form, were enrolled in the study. After standard noninvasive and invasive investigation patients classified into Group 1: PAH, (*n* = 252)) included 116 with idiopathic, heritable, or drug-induced PAH; 74 with PAH associated with congenital heart disease; 50 with PAH associated with connective tissue disease; 3 with PAH associated with hereditary hemorrhagic telangiectasia; 2 with PAH with features of venous/capillary involvement (PVOD/PCH); 6 with portopulmonary hypertension; and 1 with PAH associated with HIV infection. Group 4: Pulmonary hypertension associated with pulmonary artery obstructions) comprised 138 patients with CTEPH.

Patients were assigned to specific groups according to the clinical classification of pulmonary hypertension, which was subsequently updated in accordance with the 2022 ESC/ERS Guidelines for the diagnosis and treatment of pulmonary hypertension. No patients in this cohort had a mean pulmonary artery pressure below 25 mm Hg, nor did any cases require reclassification following the release of the new ESC/ERS guidelines (5).

Most patients self-identified as being of European ancestry. Routine measurement of protein C, protein S, and antithrombin activity was not performed because patients were receiving long-term anticoagulant therapy (6). Whole-blood samples were collected by venipuncture into EDTA tubes at the time of first hospitalization. The unopened tubes were frozen and stored in the own local PH biobank at −40 to −60 °C until DNA extraction.

### 2.2 Sequencing

Genomic DNA was isolated using a magnetic bead–based extraction approach with the MGI MGIEasy Magnetic Beads Blood Genomic DNA Extraction Kit and subsequently used for genomic library construction. Sequencing libraries were generated using a PCR-free workflow incorporating enzymatic fragmentation of DNA with the MGI MGIEasy FS PCR-Free Library Prep Set (96 reactions, MIX). Paired-end sequencing (PE150) was carried out on the DNBSEQ-T7 platform according to the manufacturer's protocols. Whole-genome sequencing was performed by Biotech Campus LLC, Moscow, Russia.

### 2.3 Variant calling and annotation

Primary processing and adapter trimming were performed using Trimmomatic (7). Reads were aligned to the GRCh38 reference genome (including alternative contigs) using the BWA-MEM algorithm (v0.7.17) in accordance with the GATK Best Practices (8). Duplicates were marked using GATK MarkDuplicatesSpark (v4.3.0.0) (9). Single nucleotide variants (SNVs) and short insertions/deletions (indels) were called using the DeepVariant tool (v1.4.0) (10). Variants that passed the PASS quality filter were included in the final analysis. For variants with QUAL < 20 or read depth (DP) < 20, manual verification of the tracks was performed using the Integrative Genomics Viewer (IGV).

Annotation of the identified variants was performed using the Variant Effect Predictor (VEP) (11). Clinical interpretation of the variants was performed according to the ACMG/AMP criteria (12) using the GeneBe service (13). Pathogenic and likely pathogenic (P/LP) variants, as well as variants of uncertain significance (VUS) with a MAF < 0.1% according to gnomAD, were included in the study.

Our primary gene set comprised genes associated with inherited thrombophilia (*PROC, PROS*1*, SERPINC*1*, F*2*, F*5*, ADAMTS*13*, HRG, JAK*2*, PIGA, THBD, F*9) that were included in the lists recommended by the ClinGen Hemostasis/Thrombosis Gene Curation Expert Panel (14) and the ISTH (https://www.isth.org/page/GinTH_GeneLists). An extended gene set additionally included *FGA, FGB, FGG*, and *CALR*, based on previously reported associations with thrombotic disorders (15, 16).

The final list included pathogenic (P) and likely pathogenic (LP) variants according to ACMG/AMP criteria, as well as variants of uncertain significance (VUS) with a minor allele frequency (MAF) < 0.1% (according to gnomAD). The use of this threshold is grounded in contemporary population genetics standards for clinical genome interpretation (17). The same MAF cutoff of <0.1% is routinely applied in large-scale international cohort studies of thrombophilia genetics, including the analysis of rare variant burden in the PROS1 gene using the UK Biobank and All of Us (18), as well as in diagnostic exome sequencing screening of patients with venous thromboembolism (19). We also detected rare variants with MAF = 0.

To ensure correct integration of genetic data into a meta-analysis alongside laboratory-confirmed coagulation factor deficiencies, we verified that each selected VUS was reported to have in silico support and/or structural and functional analysis (detailed annotation is provided in Supplementary Table S1).

### 2.4 Polygenic risk score

To calculate the polygenic risk score (PRS), we used scales developed for estimating the risk of venous thromboembolism (PGS000043 (20), PGS001796 (21), PGS002235 (22), PGS002772 (23), PGS002794 (24), PGS003332 (25), PGS003456 (26), PGS004501 (27), PGS004853 (28) and PGS004854 (28). The ancestry composition of the discovery cohorts underlying each PRS was assessed based on the information provided in the PGS Catalog. Most of the evaluated PRS were developed using predominantly European-ancestry discovery cohorts, whereas several PRS (PGS000043, PGS001796, PGS002235, PGS002772, and PGS003456) were derived from multi-ancestry discovery cohorts with European-ancestry individuals constituting the predominant component.

### 2.5 Systematic review and meta-analysis

A systematic search of the PubMed database was performed using the query “(chronic thromboembolic pulmonary hypertension OR CTEPH) AND (protein C deficiency OR protein S deficiency OR antithrombin deficiency OR factor V Leiden OR prothrombin G20210A OR thrombophilia)” (search date: 24.03.2026). In addition to the database search, the reference lists of the identified studies and relevant review articles were manually screened to identify additional relevant publications. Study selection and data extraction were performed independently by two reviewers, with results subsequently cross-checked. Discrepancies were resolved by consensus. We included original studies (prospective or retrospective cohorts) with consecutive enrollment of CTEPH patients tested for antithrombin, protein S, and protein C deficiencies, and *F*2 G20210A and *F*5 Leiden variants. Reports describing other types of pulmonary hypertension or lacking data on thrombophilia were excluded. No language restrictions were applied. The following data were extracted from each study: year of publication, the total number of patients tested for each thrombophilic defect, and the number of patients identified with these deficiencies or variants. A formal protocol was not registered for this review. The quality of studies and certainty of evidence were ensured by including only prospective or retrospective cohorts with consecutive enrollment, which minimized selection bias. Two reviewers independently assessed the methodological quality and risk of bias of the included prevalence studies using the Joanna Briggs Institute (JBI) Critical Appraisal Checklist (29). The assessment considered the representativeness and sampling of the CTEPH population, the ascertainment and standardization of thrombophilia testing, the completeness of testing and reporting, and the appropriateness of the prevalence estimation. Detailed assessments are provided in Supplementary Table S18.

The meta-analysis was conducted in accordance with the PRISMA 2020 statement (30) and MOOSE guidelines (31). The Freeman-Tukey transformation was used to calculate the pooled weighted proportion (prevalence) and 95% confidence intervals (CI) under both fixed and random effects models. Heterogeneity was assessed using the Q statistic and the *I*^2^ index. In cases of substantial heterogeneity (*I*^2^ > 75%), the random effects model was applied, while the fixed effects model was used for lower heterogeneity levels. For the Q-statistic, a *p* < 0.1 was considered to indicate significant heterogeneity. The presence of publication bias was assessed using Egger's and Begg's tests.

### 2.6 Statistical analysis

Nominal variables were compared between the two groups using the Chi-squared test, and continuous variables were compared using the Mann–Whitney *U* test. A *p*-value < 0.05 was considered statistically significant. Data processing was performed using R, Medcalc (v22.023), and SPSS (v27.0).

Raw PRS values were calculated as the weighted sum of risk alleles across the respective sets of genetic variants using the following formula:

$$PRSij\;=\sum_{k}\left(\beta k*Gik\right)$$

where *β*k is the effect weight of the k-th variant defined in the original PRS model, and Gik is the number of effect alleles (0, 1, or 2) for the i-th individual (32).

### 2.7 Machine learning models

Machine learning models were constructed using the AutoGluon automated machine learning framework (33) was used with internal 5-fold cross-validation, an 80%–20% train-test split, and bootstrap analysis (300 iterations). Variables with more than 5% missing values in the training sample were excluded; no imputation was performed, and only complete-case observations were used for each model. Polygenic risk scores were z-score standardized based on the training sample prior to inclusion. No additional feature scaling was required, as AutoGluon applies StandardScaler automatically for linear model components and tree-based methods do not require normalisation.

AutoGluon constructs a multi-layer stack ensemble rather than selecting a single optimal model. In the first layer, a diverse set of base algorithms is trained in parallel, including gradient boosting methods (LightGBM, CatBoost, XGBoost), random forests, neural networks, and k-nearest neighbours, each fitted with bagged K-fold cross-validation to reduce overfitting. In subsequent layers, out-of-fold predictions from the best-performing base models are appended to the original feature set, and new models are trained on this enriched input. A weighted ensemble at the final layer assigns weights to all models across all layers in proportion to their validation performance.

AutoGluon in automated machine learning mode with the best quality preset, which entails a broad search across a wide range of models and hyperparameters, including ensembling via bagging and stacking. The specific hyperparameters of the base models were not rigidly fixed; rather, they were adaptively tuned by AutoGluon to our dataset. Typical ranges for LightGBM include num leaves from 8 to 64 (often set to 31), learning_rate 0.03–0.1, feature_fraction 0.7–1.0, bagging_fraction 0.7–1.0, min_child_weight 1 × 10^−3^ to 10, and up to 1,000 iterations with early stopping; for CatBoost, typical values are depth 4–10, learning_rate 0.01–0.1, l2_leaf_reg 1–10, and iterations up to 1,000; for XGBoost—max_depth 3–10, learning_rate 0.01–0.3, subsample 0.6–1.0, and colsample bytree 0.6–1.0; and for Random Forest—n_estimators up to 300 and max_depth up to 20. The final predictive model is a weighted ensemble of several heterogeneous algorithms; consequently, there is no single fixed number of parameters—each base model has its own parameter count, and the total depends on the selected hyperparameters and the size of the training sample. In clinical prediction tasks, we deliberately do not emphasize the number of parameters, as the primary value of the model resides in its interpretability and predictive quality (AUC), rather than in the sheer number of weights.

The most informative clinical features with non-zero importance were identified using feature importance analysis.

Polygenic risk scores (PRS) were analyzed both separately from and in combination with clinical features. Comparison between the PRSs was performed based on the ranking results of raw discriminant analysis followed by an evaluation of their discriminative ability in machine learning models.

Results obtained with AutoGluon (an AutoML approach) were also validated using univariate and multivariate regression analyses.

## 3 Results

### 3.1 Genetic variants associated with thrombosis risk

Among the 60 patients carrying thrombophilia-associated genetic variants, 19 (7.5%) were identified in the PAH group and 41 (29.7%) in the CTEPH group. In the PAH group, pathogenic or likely pathogenic variants in SERPINC1, PROS1, and F2 G20210A were identified in 2 (0.8%), 1 (0.4%), and 11 (4.4%) patients, respectively, while F5 Leiden variants were identified in 5 (2.0%) patients. A variant of uncertain significance (VUS) in SERPINC1 was identified in 1 (0.4%) patient. In the CTEPH group, pathogenic or likely pathogenic variants were identified in SERPINC1 in 4 (2.9%) patients, PROS1 in 2 (1.4%), PROC in 4 (2.9%), JAK2 in 1 (0.7%), CALR in 1 (0.7%), and FGG in 1 (0.7%) patient. VUS were identified in SERPINC1 in 1 (0.7%), PROS1 in 3 (2.2%), and PROC in 2 (1.4%) patients. F5 Leiden and F2 G20210A variants were observed in 8 (5.8%) and 14 (10.1%) CTEPH patients, respectively, compared with 5 (2.0%) and 11 (4.4%) PAH patients, respectively (Table 1).

**Table 1:** Variants in thrombosis-associated genes in PAH and CTEPH patients.

Characteristic	PAH (*n* = 252)	CTEPH (*n* = 138)	*p*-value (Chi-squared test)
SERPINC1 P/LP, *n* (%)	1 (0.4)	4 (2.9)	0.036
SERPINC1 VUS, *n* (%)	1 (0.4)	1 (0.7)	0.665
PROS1 P/LP, *n* (%)	1 (0.4)	2 (1.4)	0.255
PROS1 VUS, *n* (%)	0 (0)	3 (2.2)	0.019
PROC P/LP, *n* (%)	0 (0)	4 (2.9)	0.007
PROC VUS, *n* (%)	0 (0)	2 (1.4)	0.055
JAK2 P/LP, *n* (%)	0 (0)	1 (0.7)	0.176
CALR P/LP, *n* (%)	0 (0)	1 (0.7)	0.176
FGG P/LP, *n* (%)	0 (0)	1 (0.7)	0.176
F5 Leiden, *n* (%)	5 (2.0)	8 (5.8)	0.045
F2 G20210A, *n* (%)	11 (4.4)	14 (10.1)	0.026

Notably, the pathogenic/likely pathogenic SERPINC1 variant rs2227624 was identified in two different patients with CTEPH. In addition, one CTEPH patient carried two variants simultaneously, namely the PROC variant rs778063605, classified as a VUS, and F2 G20210A. The VUS were included due to their low population frequency, the absence of homozygotes in population databases, and their presence in previously described patients with coagulation factor deficiencies. Overall, 59 distinct genetic variants were identified among the 60 patients, with rs2227624 representing the only variant identified in more than one patient (Supplementary Table S1).

No cases of double heterozygosity, compound heterozygosity, or homozygosity involving F5 Leiden and F2 G20210A were observed in the studied cohort.

### 3.2 Prevalence of hereditary thrombophilias in CTEPH: a meta-analysis

We identified 80 publications. Of these, 28 studies were selected based on their titles and 23 based on their abstracts. A total of 15 studies containing information on hereditary thrombophilia testing were included in the final analysis (Supplementary Table S2, Supplementary Figure S1). The main reasons for exclusion were a lack of data on thrombophilia testing or a focus on non-CTEPH patients. The included studies were published between 1995 and 2025. All 15 studies were assessed as having a low risk of selection bias due to the consecutive enrollment of patients, consistent with our inclusion criteria.

In previously published studies genotyping was not performed in most cases, and the correlation of genetic findings with clinical phenotype was presented in only one study (34). To include our group's results in the overall calculation, the carriage of the identified variants was considered equivalent to laboratory factor deficiency (despite the lack of biochemical verification, which is a study limitation).

Antithrombin deficiency: 11 studies (including 3,008 patients) were included regardless of the region. Due to moderate heterogeneity, a fixed effects model was used. The prevalence was 3.0% [95% CI: 2.4–3.6] (Supplementary Tables S3–S5, Supplementary Figure S2).

Protein S deficiency: 11 studies (including 3,244 patients) were included regardless of the region. Due to high data heterogeneity, a random effects model was used. The prevalence was 3.6% [95% CI: 1.8–5.9] (Supplementary Tables S6–S8, Supplementary Figure S3).

Protein C deficiency: 12 studies (including 3,262 patients) were included regardless of the region. Due to high data heterogeneity, a random effects model was used. The prevalence was 4.7% [95% CI: 3.0–6.6] (Supplementary Tables S9–S11, Supplementary Figure S4).

*F*5 Leiden: 9 studies (including 759 patients) involving only genotyping data were included. Studies conducted in the East Asian region were excluded due to low population carrier frequency. Due to moderate heterogeneity, a fixed effects model was used. The prevalence was 7.9% [95% CI: 6.1–10.1] (Supplementary Tables S12–S14, Supplementary Figure S5).

*F*2 G20210A: 10 studies (including 1,179 patients) were included (studies from East Asia were excluded). Due to moderate heterogeneity, a fixed effects model was used. The prevalence was 5.6% [95% CI: 4.3–7.1] (Supplementary Tables S15–S17, Supplementary Figure S6).

No evidence of publication bias was found during the meta-analyses.

Visual analysis of the funnel plots and forest plots (Figure 1) demonstrates the absence of critical deviations in our patient group. The detection rate of hereditary thrombophilias in our study was: antithrombin deficiency—3.6% [1.2–8.3], protein S deficiency—3.6% [1.2–8.3], protein C deficiency—4.3% [1.6–9.2], *F*5 Leiden—5.8% [2.5–11.1], and *F*2 G20210A—10.1% [5.7–16.4]. These values fall within the confidence intervals of the conducted meta-analyses (Supplementary Tables S3–S17, Supplementary Figures S2–S6).

**Figure 1:**
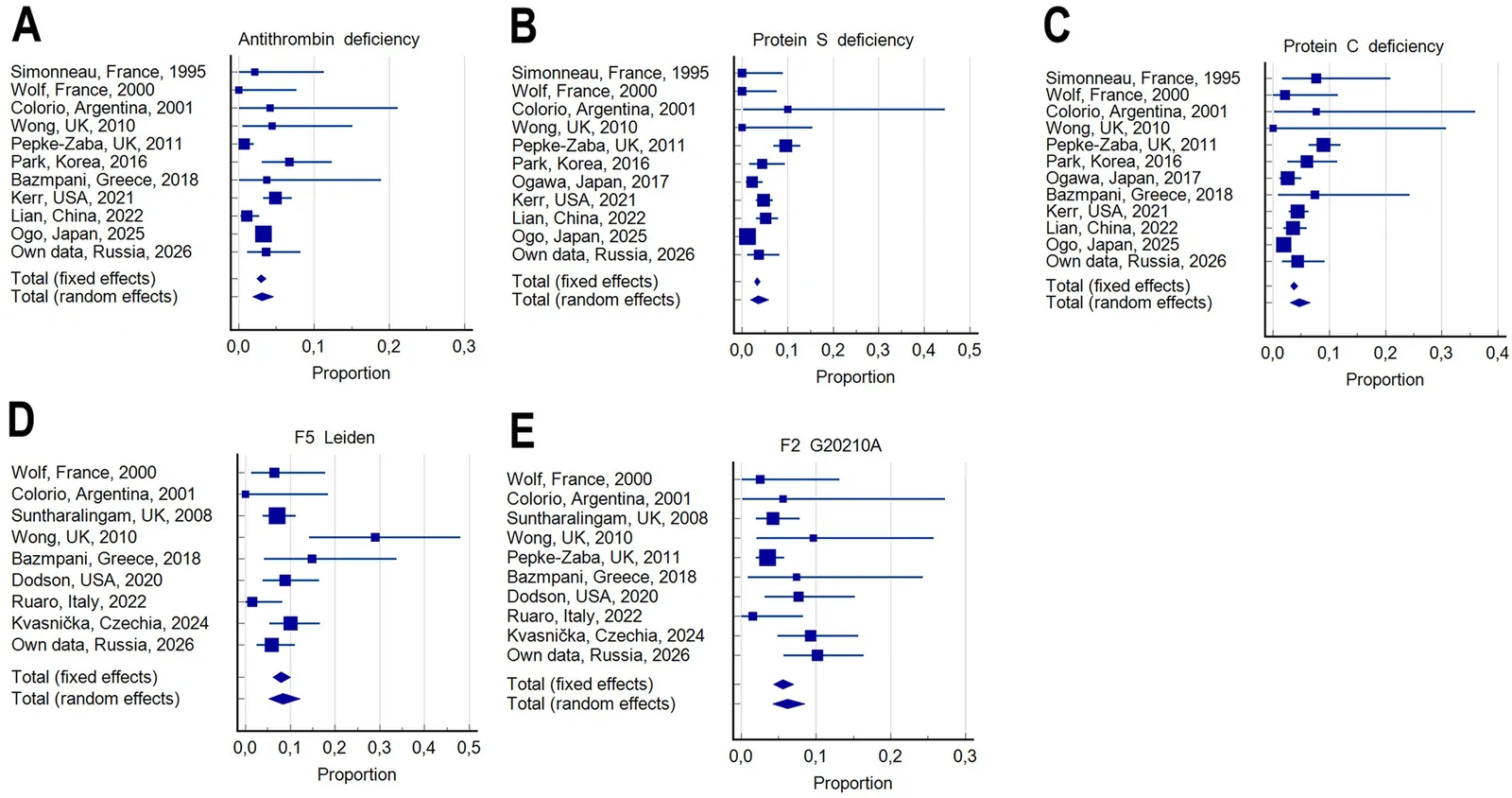
Forest plots for the meta-analyses of hereditary thrombophilia prevalence in patients with CTEPH. **(A)** Antithrombin deficiency. **(B)** Protein S deficiency. **(C)** Protein C deficiency. **(D)** *F*5 Leiden*.* **(E)** *F*2 G20210A.

### 3.3 Analysis of clinical features, polygenic risk scores, and rare variants in thrombosis-associated genes in the differential diagnosis of CTEPH

We applied machine learning approach to classify CTEPH and PAH patients using different groups of features—clinical, polygenic risk scores and rare variants in thrombosis-associated genes—both alone and in combination. First, we built machine learning models using only clinical characteristics (Table 2). Using AutoGluon, an AutoML approach, we trained a model that achieved an ROC AUC = 0.883 and identified 20 clinical features as the most important for the differential diagnosis of CTEPH. In all subsequent analyses, any machine learning model that included clinical features was built using this set of 20 clinical variables.

**Table 2:** Patient characteristics.

Characteristic		PAH (*n* = 252)	CTEPH (*n* = 138)	*p*-value
Males/females, *n* (%)		52 (20.6)/200 (79.4)	53 (38.4)/85 (61.6)	<0.001
Age at enrollment, years		51.9 ± 15.82	63.9 ± 13.50	<0.001
Age at onset of PH symptoms, years		44.8 ± 19.97	60.6 ± 14.52	<0.001
Age at PH diagnosis, years		48.5 ± 18.74	63.0 ± 13.79	<0.001
WHO Functional Class	I, *n* (%)	6 (2.4)	2 (1.4)	0.029
	II, *n* (%)	65 (25.8)	19 (13.8)	
	III, *n* (%)	170 (67.5)	107 (77.5)	
	IV, *n* (%)	11 (4.4)	10 (7.2)	
Lower extremity edema, *n* (%)		108 (42.9)	68 (49.3)	0.223
Syncope, *n* (%)		46 (18.3)	18 (13.0)	0.184
Raynaud's phenomenon, *n* (%)		42 (16.7)	1 (0.7)	<0.001
6MWD, m		377.0 ± 115.35	338.5 ± 91.16	0.004
NT-proBNP, pg/ml		2,262 [306; 3,060]	2,943.0 [471; 3,775]	0.161
Anthropometric data				
Height, cm		164.2 ± 8.85	168.3 ± 9.89	<0.001
Weight, kg		75.9 ± 20.56	81.4 ± 19.74	0.010
Body mass index, kg/m²		28.1 ± 7.50	28.7 ± 6.31	0.233
Body surface area, m²		1.81 ± 0.239	1.90 ± 0.244	<0.001
Echocardiography				
LVEF, %		60.4 ± 4.93	59.9 ± 6.01	0.153
sPAP, mmHg		77.5 ± 22.67	71.4 ± 21.45	0.015
TAPSE, mm		17.5 ± 4.46	17.0 ± 4.47	0.519
Pericardial effusion, *n* (%)		88 (34.9)	37 (26.8)	0.101
Right heart catheterization				
mPAP, mmHg		51.2 ± 16.65	46.1 ± 13.27	0.019
PVR, Wood units		10.2 ± 5.89	9.4 ± 5.38	0.399
CI, L/min/m²		2.6 ± 1.22	2.4 ± 1.03	0.237
mRAP, mmHg		8.3 ± 5.20	8.8 ± 5.96	0.802
Risk factors and comorbidities				
Smoking, *n* (%)		33 (13.1)	33 (24.3)	0.005
Hypertension, *n* (%)		148 (58.7)	110 (79.7)	<0.001
Known atherosclerosis, *n* (%)		44 (17.5)	56 (40.6)	<0.001
History of myocardial infarction, *n* (%)		6 (2.4)	18 (13.0)	<0.001

6MWD, 6-minute walk distance; mPAP, mean pulmonary artery pressure; PVR, pulmonary vascular resistance; CI, cardiac index; mRAP, mean right atrial pressure; LVEF, left ventricle ejection fraction; sPAP, systolic pulmonary artery pressure.

Next, we constructed Machine learning models for each PRS scale separately using only polygenic scores (Table 3, Table 4). This approach allowed us to assess the independent contribution of each polygenic score to disease classification prior to its integration into models that included clinical features and thrombophilia variant carriage.

**Table 3:** Discriminative ability of raw PRS across patient groups.

Scale	PAH (*n* = 252)	CTEPH (*n* = 138)	*p*-value
PGS004854 (28)	0.0403 ± 0.2399	0.1835 ± 0.2889	<0.001
PGS004853 (28)	−0.0741 ± 0.2021	0.0439 ± 0.2485	<0.001
PGS002772 (23)	−0.1996 ± 0.0790	−0.1598 ± 0.0834	<0.001
PGS004501 (27)	−0.1046 ± 0.1363	−0.0549 ± 0.1169	<0.001
PGS002794 (24)	1.0766 ± 0.4327	1.2851 ± 0.5606	<0.001
PGS002235 (22)	0.3608 ± 0.1941	0.4278 ± 0.2066	0.002
PGS000043 (20)	9.0248 ± 1.4969	9.3959 ± 1.6793	0.031
PGS003456 (26)	8.7479 ± 1.4142	9.0675 ± 1.5713	0.048
PGS001796 (21)	−0.2394 ± 0.1300	−0.2268 ± 0.1427	0.389
PGS003332 (25)	−0.1048 ± 0.0554	−0.1137 ± 0.0571	0.142

**Table 4:** Machine-learning models based on clinical features, polygenic risk scores and variants associated with thrombophilias.

Scale	Clinical variables	Hereditary thrombophilia	PRS	Clinical variables + rare variants in thrombosis-associated genes
Only pathogenic and likely pathogenic rare variants in thrombosis-associated genes	0.878 ± 0.027			
Clinical variables only	0.883 ± 0.025			
Any hereditary thrombophilia	0.918 ± 0.032	0.680 ± 0.049		
Rare variants in thrombosis-associated genes	0.909 ± 0.037	0.582 ± 0.029		
F5 Leiden and F2 G20210A	0.895 ± 0.043	0.598 ± 0.044		
PGS004854 (28)	0.928 ± 0.038		0.698 ± 0.066	**0.951** ± **0.022**
PGS002772 (23)	0.893 ± 0.043		0.606 ± 0.067	0.923 ± 0.030
PGS004853 (28)	0.922 ± 0.038		0.675 ± 0.067	0.928 ± 0.035
PGS002794 (24)	0.863 ± 0.045		0.498 ± 0.066	0.874 ± 0.041
PGS004501 (27)	0.904 ± 0.041		0.585 ± 0.067	0.924 ± 0.031
PGS000043 (20)	0.891 ± 0.043		0.559 ± 0.072	0.923 ± 0.031
PGS001796 (21)	0.881 ± 0.045		0.523 ± 0.062	0.900 ± 0.038
PGS002235 (22)	0.889 ± 0.041		0.536 ± 0.063	0.903 ± 0.035
PGS003332 (25)	0.883 ± 0.042		0.458 ± 0.064	0.893 ± 0.036
PGS003456 (26)	0.896 ± 0.040		0.477 ± 0.067	0.920 ± 0.033

The addition of PRS to the clinical model led to changes in discriminative ability in several cases compared to the model based on clinical features alone; however, no statistically significant differences were obtained. The best performance was achieved with scales PGS004854 (AUC: 0.928 ± 0.038), PGS004853 (AUC: 0.922 ± 0.038), and PGS004501 (AUC: 0.904 ± 0.041).

Given that the F5 Leiden variant is a constituent of most PRS (PGS001796, PGS002235, PGS002772, PGS002794, PGS003332, PGS004501, PGS004853, and PGS004854) and the F2 G20210A variant is included in four of them (PGS002235, PGS002794, PGS004853, and PGS004854), we exclude these SNPs from the analysis in models due to inherent redundancy and the risk of collinearity.

The PGS004854 (28) consistently demonstrated the highest discriminative ability, maintaining its advantage across standalone PRS models, combined models with clinical features and thrombophilias, as well as in univariate and multivariate analyses (Figure 2).

**Figure 2:**
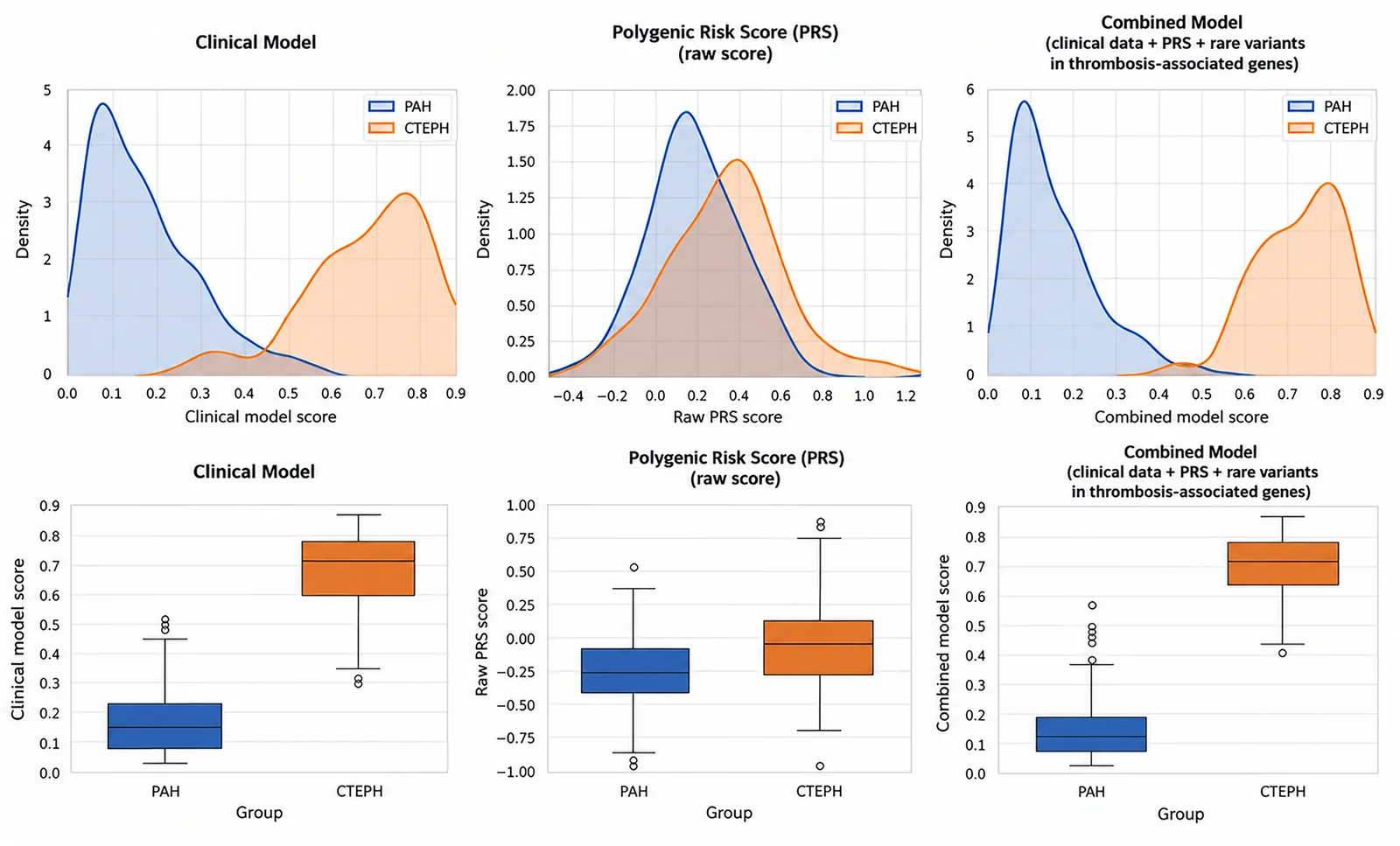
Predicted probability of CTEPH and PAH patients by models based on clinical features, raw PRS scores and combined model integrating clinical, PRS, and rare variants in thrombosis-associated genes.

Third, we built models based solely on the presence of variants associated with hereditary thrombophilias. Separate models were built for rare variants in thrombosis-associated genes and for F5 Leiden and F2 G20210A variants, as well as a model combining all hereditary thrombophilias. These models demonstrated low, though significant, predictive value (Table 4). To evaluate the discriminative value of these groups, each was individually integrated into the clinical model (Table 4). This integration improved the model's discriminative ability, yielding ROC AUC = 0.895 for clinical plus F5 Leiden and F2 G20210A model, 0.909 for clinical plus rare variants in thrombosis-associated genes, and 0.918 for clinical plus all variants.

When clinical features, PRS scales, and rare variants in thrombosis-associated genes were combined into integrated models, the ROC AUC values ranged from 0.893 ± 0.036 to 0.951 ± 0.022 across different scales (Table 4). The highest classification accuracy was achieved by the model combining clinical model, the PGS004853 scale, and rare variants in thrombosis-associated genes.

Adding the most effective polygenic score (PGS004854) and rare variants in thrombosis-associated genes data to the baseline clinical model resulted in a 7.7% increase in the mean ROC AUC value, which was statistically significant (DeLong's test for comparing the ROC AUC of the clinical and combined models, *p* = 0.026).

Additionally, we tested a model that included clinical features along with only pathogenic and likely pathogenic variants in thrombosis-associated genes. This configuration yielded a ROC AUC of 0.878 ± 0.027, which did not exceed the performance of the clinical model without genetic data (AUC: 0.883 ± 0.025).

### 3.4 Univariate and multivariate logistic regression analysis

Here we applied univariate and multivariate analyses to 20 clinical features and PRS scales (Table 5). According to the univariate analysis results, statistically significant associations with CTEPH were demonstrated by Raynaud's phenomenon, body surface area, history of myocardial infarction, known atherosclerosis, hypertension, smoking, male sex, age at symptom onset, lower extremity edema, systolic pulmonary artery pressure, and creatinine. Notably, the polygenic score PGS004854 showed the highest positive odds ratio for CTEPH detection (OR = 6.112; 95% CI: 2.379–15.701; *p* < 0.001).

**Table 5:** Univariate and multivariate analysis. Odds ratios for CTEPH detection.

Feature	Univariate analysis		Multivariate analysis		
	OR [95% CI]	*P* value	OR [95% CI]	*P* value	*β* coefficient
Raynaud's phenomenon	0.041 [0.006–0.304]	0.002	0.024 [0.003–0.217]	<0.001	−3.719
PGS004854 (28)	6.112 [2.379–15.701]	<0.001	6.485 [1.757–23.933]	<0.001	1.869
Body surface area	4.638 [1.699–12.658]	0.003	2.789 [0.549–14.165]	0.216	1.026
History of myocardial infarction	5.954 [2.084–17.011]	<0.001	2.394 [0.572–10.020]	0.232	0.873
Diabetes mellitus	1.104 [0.602–2.025]	0.748	0.454 [0.199–1.037]	0.061	−0.789
Known atherosclerosis	3.048 [1.824–5.094]	<0.001	1.865 [0.832–4.179]	0.130	0.623
Syncope	0.597 [0.309–1.151]	0.123	0.617 [0.247–1.543]	0.302	−0.483
Arterial hypertension	2.204 [1.302–3.731]	0.003	0.616 [0.242–1.564]	0.308	−0.485
Smoking	2.266 [1.260–4.075]	0.006	1.796 [0.788–4.097]	0.164	0.586
History of vascular stenting	2.653 [0.896–7.854]	0.078	0.556 [0.121–2.547]	0.450	−0.587
Pericardial effusion	0.662 [0.394–1.111]	0.118	0.560 [0.267–1.178]	0.127	−0.579
Male sex	2.211 [1.322–3.698]	0.002	1.541 [0.638–3.723]	0.337	0.432
Absolute leukocyte count	1.049 [0.955–1.152]	0.322	1.196 [1.033–1.384]	0.016	0.179
Age at symptom onset	1.051 [1.034–1.068]	<0.001	1.072 [1.043–1.101]	<0.001	0.069
Erythrocyte count	0.835 [0.614–1.137]	0.253	1.039 [0.558–1.936]	0.904	0.038
Edema of lower extremities	1.614 [1.001–2.601]	0.049	0.911 [0.451–1.840]	0.795	−0.093
Systolic pulmonary artery pressure	0.984 [0.973–0.996]	0.006	0.991 [0.974–1.008]	0.284	−0.009
Hemoglobin level	0.993 [0.983–1.004]	0.206	0.993 [0.973–1.014]	0.506	−0.007
Creatinine	1.011 [1.002–1.020]	0.021	1.006 [0.991–1.021]	0.453	0.006
Systolic blood pressure	1.000 [0.987–1.014]	0.968	0.997 [0.976–1.018]	0.767	−0.003
Platelet count	1.002 [0.998–1.005]	0.323	0.999 [0.994–1.003]	0.534	−0.001

In the multivariate analysis, statistical significance was achieved for the following variables: Raynaud's phenomenon, absolute leukocyte count, and age at symptom onset. The polygenic risk score (PRS) provided a substantial contribution among all included factors (*β* = 1.869). Consequently, the multivariate logistic regression model yielded an AUC of 0.898 ± 0.045, *p* < 0.001.

## 4 Discussion

This study presents three key findings. First, we document the prevalence of hereditary thrombophilias in a Russian cohort of CTEPH patients and demonstrate its consistency with data reported from other populations. Second, we show that the genetic profile of CTEPH patients differs significantly from that of PAH patients, with hereditary thrombosis risk factors occurring at substantially higher frequencies. Third, we demonstrate that integrating polygenic risk scores and rare variants in thrombosis-associated genes into clinical diagnostic models significantly improves differential diagnosis accuracy, with the best-performing model achieving ROC AUC of 0.951.

### 4.1 Hereditary thrombophilia in CTEPH: prevalence and comparison with other populations

The prevalence of hereditary thrombophilias in our CTEPH cohort is consistent with the meta-analytic estimates derived from 15 independent studies. This cross-population consistency is noteworthy given that the present study constitutes, to our knowledge, the first systematic investigation of hereditary thrombophilia prevalence in Russian patients with CTEPH.

The significantly higher prevalence of both rare variants in thrombosis-associated genes (12.3% vs. 1.2%, *p* < 0.001) and F5 Leiden/F2 G20210A variants (15.9% vs. 6.3%, *p* = 0.002) in CTEPH compared to PAH patients supports the established concept that hereditary prothrombotic conditions contribute to the pathogenesis of CTEPH. This finding aligns with prior observations from registry-based and case-control studies, which have consistently identified thrombophilic states as risk factors for CTEPH, even though they are not regarded as primary drivers of the transition from acute pulmonary embolism to the chronic disease state. The particular enrichment for protein C deficiency (*PROC* variants in 4.3% of CTEPH vs. 0% in PAH) and protein S deficiency (3.6% vs. 0.4%) is consistent with the known roles of these natural anticoagulants in thrombus resolution and fibrinolysis. Impaired clot lysis due to deficiency of these factors may facilitate the persistence and organization of thromboembolic material, ultimately leading to the chronic occlusive lesions characteristic of CTEPH.

The critical appraisal of the included studies highlights important methodological caveats in the existing literature on hereditary thrombophilias in CTEPH. While the risk of selection bias was consistently low—indicating that the sampled populations accurately reflect CTEPH cohorts—the overall methodological quality was often compromised by historical reporting practices and inherent clinical challenges. Specifically, the statistical power for evaluating rare thrombophilias was frequently insufficient due to small cohort sizes. Furthermore, the validation and standardization of diagnostic methods were clearly documented in only a minority of studies. This is largely attributable to the era in which older studies were conducted and the well-known difficulty of performing functional biochemical assays (particularly for protein C, protein S, and antithrombin) in patients receiving lifelong anticoagulant therapy. Consequently, the pooled prevalence rates derived from our meta-analysis represent the best available evidence but must be interpreted with an understanding of these historical limitations. These findings underscore a critical gap in the literature: there is an urgent need for large, prospective, multicenter registries that utilize standardized, modern diagnostic algorithms—ideally combining both functional assays and comprehensive genetic sequencing—to definitively establish the epidemiological and clinical burden of hereditary thrombophilias in CTEPH.

An important methodological consideration concerns the classification of variants of uncertain significance in this study. We included VUS with a minor allele frequency < 0.1% in gnomAD that had in silico and/or structural-functional support, an approach consistent with contemporary population genetics standards for clinical genome interpretation and with large-scale studies of thrombophilia genetics in the UK Biobank and All of Us cohorts. While this approach may introduce some degree of misclassification, it represents a pragmatic solution to the inherent limitations of laboratory phenotyping in patients on long-term anticoagulation—a context in which measurement of protein C, protein S, and antithrombin activity is unreliable. It should be noted that genetic testing for these deficiencies is not routinely performed, and few studies have simultaneously utilized both genotyping and functional assays in CTEPH. Moreover, an analysis that automatically excludes all variants of uncertain significance (VUS) represents an overly conservative and rigid approach in the context of exploratory genetic research. Such an approach may lead to the exclusion of variants that, upon receipt of additional data in the future, could prove to be biologically or clinically meaningful. In our study, this is particularly relevant for variants identified in thrombophilia-associated genes, which have established roles in inherited thrombophilia. Furthermore, the predominance of VUS among patients with CTEPH compared with PAH (7 vs. 1) provides additional rationale for considering these variants as potential indicators of increased thrombotic susceptibility. Notably, some of these VUS have previously been reported in association with laboratory-confirmed thrombophilia and/or thrombotic events in carriers [NM_000313.4:c.814G > A (35); NM_000313.4:c.1331C > T (36); NM_000488.4:c.529C > T (37).

The observed predictive value of these variants should therefore be interpreted as a model-level research association reflecting the presence of rare genetic variation in thrombophilia-associated genes, rather than as definitive evidence of the pathogenicity of individual variants.

Our literature review identified only one such study, which reported that 20%–75% of patients with laboratory-confirmed deficiencies carried pathogenic or likely pathogenic variants in the corresponding genes (34). Consequently, for our meta-analysis of these markers, we did not limit the search to genetic studies but included all works, most of which relied on functional laboratory methods. A different approach was applied to activated protein C resistance. While this can be determined via *F*5 Leiden genotyping or functional tests (with 90%–95% concordance), the abundance of genetic data available (nine studies, *n* = 621) allowed us to apply stricter criteria. Therefore, we excluded studies that relied solely on functional assays without genotyping (resulting in the exclusion of two studies, *n* = 978 (38, 39). Of note, VUS comprised only 33.3% of identified rare variants in thrombosis-associated genes, with the majority being classified as pathogenic or likely pathogenic by ACMG/AMP criteria. Nevertheless, the absence of biochemical verification remains a study limitation, and may introduce potential heterogeneity; thus, future studies incorporating paired genetic and functional data will be important to validate these classifications.

### 4.2 Polygenic risk scores in the differential diagnosis of CTEPH

The application of polygenic risk scores for venous thromboembolism to the differential diagnosis of CTEPH represents a conceptual advancement in this field. Among the ten PRS evaluated, PGS004854 and PGS004853—both derived from the large-scale genome-wide association analysis of VTE—demonstrated the highest discriminative ability, both as standalone predictors and in combination with clinical features. PGS004854 was the strongest positive genetic predictor of CTEPH in multivariate logistic regression (*β* = 1.869, OR = 6.485, *p* < 0.001), underscoring its independent contribution beyond clinical variables alone. An important consideration regarding the observed performance of PGS004854 is the ancestral composition of both the discovery and study populations. PGS004854 was developed primarily using genetic data from individuals of European ancestry, while our study cohort was also predominantly of European ancestry. Thus, the strong discriminative performance observed for PGS004854 may reflect, at least in part, its applicability to the ancestral background represented in our cohort. Although several of the evaluated PRS were developed using multi-ancestry discovery cohorts (PGS000043, PGS001796, PGS002235, PGS002772, and PGS003456), European-ancestry individuals constituted the predominant component of these cohorts.

This superiority of PGS004854 over other VTE risk scores likely reflects differences in the GWAS discovery cohorts, the number and weighting of included variants, and the extent to which the underlying genetic architecture of VTE pathways overlaps with CTEPH-specific mechanisms. Notably, the scales that performed less well (e.g., PGS003332, PGS002794) were either derived from smaller discovery samples or used methodological approaches that may be less transferable to non-European populations. The relatively modest performance of some PRS scales as standalone classifiers (AUC ranging from 0.458 to 0.698) confirms that polygenic scores for VTE do not in isolation provide sufficient discrimination between CTEPH and PAH, but their incremental value when added to clinical models is clinically meaningful.

The statistically significant 7.7% improvement in ROC AUC (from 0.883 to 0.951, *p* = 0.026) achieved by adding PGS004854 and rare variants in thrombosis-associated genes to the clinical model is practically relevant. A model with AUC > 0.94 can substantially reduce the number of misclassified patients—particularly false negatives (PAH patients classified as CTEPH who would then be inappropriately referred for surgical evaluation, or conversely, CTEPH patients denied timely pulmonary endarterectomy). Given the high stakes of the differential diagnosis between CTEPH and PAH—where the correct identification of CTEPH directly opens the pathway to potentially curative surgical treatment—even modest improvements in diagnostic accuracy carry meaningful clinical weight. Of note, restricting rare variants in thrombosis-associated genes input to only pathogenic and likely pathogenic variants (excluding VUS) reduced model performance below the clinical-only baseline (AUC: 0.873), further emphasizing the importance of including well-annotated VUS in genetic diagnostic panels. Nevertheless, despite the promising predictive performance demonstrated by this combined model, these findings require cautious interpretation and warrant further validation in an independent patient cohort.

### 4.3 Clinical features in the differential diagnosis of CTEPH

The machine learning clinical model alone achieved a ROC AUC of 0.883, consistent with the reported performance of clinical scoring systems for CTEPH differential diagnosis in prior studies. The most discriminative clinical features in our multivariate analysis included Raynaud's phenomenon (strongly associated with PAH, particularly in the context of connective tissue disease), age at symptom onset, and absolute leukocyte count. The inverse association of Raynaud's phenomenon with CTEPH (OR = 0.024 in multivariate analysis) reflects the much higher prevalence of connective tissue disease-associated PAH in our PAH cohort (50 of 252 patients). Conversely, the higher rates of cardiovascular comorbidities in CTEPH patients—including known atherosclerosis (40.6% vs. 17.5%), history of myocardial infarction (13.0% vs. 2.4%), and hypertension (79.7% vs. 58.7%)—reflect the older age and different risk factor profile of the CTEPH population. These findings are consistent with the recognized epidemiological overlap between CTEPH and systemic atherothrombotic disease.

The age difference between groups (mean 63.9 years in CTEPH vs. 51.9 years in PAH) is well established in the literature and reflects both the generally later onset of CTEPH and the growing recognition of PAH manifesting at older ages with multiple comorbidities. The significant differences in 6-minute walk distance (338.5 vs. 377.0 m, *p* = 0.004) and hemodynamic parameters—including lower mean pulmonary artery pressure in CTEPH (46.1 vs. 51.2 mmHg, *p* = 0.019)—suggest that CTEPH patients in our cohort were diagnosed at a stage of somewhat less severe hemodynamic compromise compared to PAH patients, possibly reflecting greater diagnostic vigilance for thromboembolic disease at our center.

### 4.4 Genetic screening in CTEPH: implications for current guidelines

Current ESC/ERS guidelines recommend genetic testing in PAH but not in CTEPH, given the absence of an identified monogenic predisposition for CTEPH analogous to that established for heritable PAH (e.g., *BMPR*2 mutations). Our findings do not challenge this framework—the genetic variants identified in CTEPH patients are not disease-causing in the classical Mendelian sense but rather represent thrombotic risk modifiers. However, they do suggest that targeted genetic screening for classical thrombophilias (*PROC, PROS*1*, SERPINC*1*, F*2*, F*5*, ADAMTS*13*, HRG, JAK*2*, PIGA, THBD, F*9*, FGA, FGB, FGG, CALR*) using whole-genome or targeted next-generation sequencing has diagnostic utility in the CTEPH population, particularly when biochemical testing is precluded by anticoagulation. Furthermore, the demonstration that polygenic risk scores can enhance differential diagnosis performance raises the prospect of incorporating PRS into future diagnostic algorithms, once prospective validation data become available.

It is also worth noting that the detection of a thrombophilic state in a CTEPH patient has direct therapeutic implications beyond diagnosis. Identification of inherited thrombophilia supports the decision for indefinite anticoagulation and may influence the choice of anticoagulant agent. In addition, the identification of at-risk family members through cascade screening could contribute to primary prevention of VTE events in relatives.

## 5 Conclusions

Hereditary thrombophilias are significantly more prevalent in patients with CTEPH than in those with PAH. The prevalence observed in this Russian cohort is consistent with data reported in other populations.

While a purely clinical model is effective for the differential diagnosis of CTEPH, the integration of rare variants in thrombosis-associated genes and the PGS004854 polygenic risk score enhances the model's predictive power by 7.7%. Despite the observed increase in predictive power, the obtained results require cautious interpretation and validation in an independent patient cohort. These findings highlight the potential of combined clinical-genetic approaches to optimize the diagnostic workup and personalize management strategies for patients with pulmonary hypertension.

## 6 Limitations

Genetic testing data do not allow for a full assessment of the thrombophilia phenotype, which may lead to an underestimation of its prevalence. Validation of the diagnostic algorithm on other cohorts of pulmonary hypertension patients, either retrospectively or in a prospective setting, was not performed. In addition, the PRS that showed the strongest association with CTEPH in our analysis (PGS004854) was developed using genetic data from individuals of European ancestry, while our study population was also predominantly of European ancestry. Therefore, the performance of PGS004854 observed in our cohort may not be generalizable to populations of non-European ancestry, and validation in more ancestrally diverse cohorts is warranted.

## Data Availability

The sequencing data of 390 PAH and CTEPH patients that support the findings of this study are not openly available due to reasons of sensitivity and are available from the corresponding author upon reasonable request. Data are located in controlled access data storage at the Centre for Biomedical Research and Technologies at HSE University. Code for the analysis performed in this study is freely available at https://github.com/Kubovski/Integration-of-thrombophilia-PRS-improves-CTEPH-differential-diagnosis.
