# Supplementary material for: Integration of hereditary thrombophilia data and polygenic risk scores into prognostic models improves differential diagnosis of CTEPH
Source: Front Cardiovasc Med. 2026 Oct 5;13:1887880. doi: 10.3389/fcvm.2026.1887880 (PMC13678304; doi:10.3389/fcvm.2026.1887880)
Supplement: Supplementary file 1 [file Datasheet2.pdf]

Supplementary Table S2. Studies containing information on hereditary thrombophilia included in the meta-analysis.

**First author. country. year**	References	Antithrombin **deficiency. n (%)**	**Protein S deficiency. n** (%)	**Protein C deficiency. n** (%)	*F5* Leiden. n (%)	*F2* G20210A. n (%)
Simonneau. France. 1995	Simonneau G. Azarian R. Brenot F. Dartevelle PG. Musset D. Duroux P: Surgical management of unresolved pulmonary embolism. A personal series of 72 patients. Chest 1995. 107(1 Suppl):52S-55S.	1/47 (2.1%)	0/39 (0%)	3/39 (7.7%)	-	-
Wolf. France. 2000	Wolf M. Boyer-Neumann C. Parent F. Eschwege V. Jaillet H. Meyer D. Simonneau G: Thrombotic risk factors in pulmonary hypertension. Eur Respir J 2000. 15(2):395-399.	0/46 (0%)	0/46 (0%)	1/46 (2.2%)	3/46 (6.5%)	1/40 (2.5%)
Colorio. Argentina. 2001	Colorio CC. Martinuzzo ME. Forastiero RR. Pombo G. Adamczuk Y. Carreras LO: Thrombophilic factors in chronic thromboembolic pulmonary hypertension. Blood Coagul Fibrinolysis 2001. 12(6):427-432.	1/24 (4.2%)	1/10 (10.0%)	1/13 (7.7%)	0/18 (0%)	1/18 (5.6%)
Suntharalingam. UK. 2008	Suntharalingam J. Goldsmith K. van Marion V. Long L. Treacy CM. Dudbridge F. Toshner MR. Pepke-Zaba J. Eikenboom JC. Morrell NW: Fibrinogen Aalpha Thr312Ala polymorphism is associated with chronic thromboembolic pulmonary hypertension. Eur Respir J 2008. 31(4):736-741.	-	-	-	15/214 (7.0%)	9/214 (4.2%)
Wong. UK. 2010	Wong CL. Szydlo R. Gibbs S. Laffan M: Hereditary and acquired thrombotic risk factors for chronic thromboembolic pulmonary hypertension. Blood Coagul Fibrinolysis 2010. 21(3):201-206.	2/45 (4.4%)	0/22 (0%)	0/10 (0%)	9/31 (29.0%)	3/31 (9.7%)
Pepke-Zaba. UK. 2011	Pepke-Zaba J. Delcroix M. Lang I. Mayer E. Jansa P. Ambroz D. Treacy C. D'Armini AM. Morsolini M. Snijder R et al: Chronic thromboembolic pulmonary hypertension (CTEPH): results from an international	3/426 (0.7%)	41/426 (9.6%)	38/426 (8.9%)	-	15/426 (3.5%)

	prospective registry. Circulation 2011. 124(18):1973-1981.					
Park. Korea. 2016	Park SY. Lee SM. Shin JW. Choi BW. Kim H. Lee JS. Lee SD. Park SS. Moon HS. Park YB: Epidemiology of chronic thromboembolic pulmonary hypertension in Korea: results from the Korean registry. Korean J Intern Med 2016. 31(2):305-312.	9/134 (6.7%)	6/134 (4.5%)	8/134 (6.0%)	-	-
Ogawa. Japan. 2017	Ogawa A. Satoh T. Fukuda T. Sugimura K. Fukumoto Y. Emoto N. Yamada N. Yao A. Ando M. Ogino H et al: Balloon Pulmonary Angioplasty for Chronic Thromboembolic Pulmonary Hypertension: Results of a Multicenter Registry. Circ Cardiovasc Qual Outcomes 2017. 10(11).	-	7/308 (2.3%)	8/308 (2.6%)	-	-
Bazmpani. Greece. 2018	Bazmpani MA. Arvanitaki A. Toumpourleka M. Pitsiou G. Panagiotidou E. Mouratoglou SA. Sianos G. Hadjimiltiades S. Pitsis A. Mayer E et al: Epidemiology and management of chronic thromboembolic pulmonary hypertension: experience from two expert centers. Hellenic J Cardiol 2018. 59(1):16-23.	1/27 (3.7%)	-	2/27 (7.4%)	4/27 (14.8%)	2/27 (7.4%)
Dodson. USA. 2020	Dodson MW. Sumner K. Carlsen J. Cirulis MM. Wilson EL. Gadre A. Fernandes TM. Brown LM. Best DH. Elliott CG: The Factor V Leiden variant and risk of chronic thromboembolic pulmonary hypertension. Eur Respir J 2020. 56(4).	-	-	-	8/91 (8.8%)	7/91 (7.7%)
Kerr. USA. 2021	Kerr KM. Elliott CG. Chin K. Benza RL. Channick RN. Davis RD. He F. LaCroix A. Madani MM. McLaughlin VV et al: Results From the United States Chronic Thromboembolic Pulmonary Hypertension Registry: Enrollment Characteristics and 1-Year Follow-up. Chest 2021. 160(5):1822-1831.	27/552 (4.9%)	26/552 (4.7%)	24/552 (4.3%)	-	-
Lian. China. 2022	Lian TY. Liu JZ. Guo F. Zhou YP. Wu T. Wang H. Li JY. Yan XX. Peng	4/367 (1.1%)	19/367 (5.2%)	13/367 (3.5%)	-	-

	FH. Sun K et al: Prevalence. Genetic Background. and Clinical Phenotype of Congenital Thrombophilia in Chronic Thromboembolic Pulmonary Hypertension. JACC Asia 2022. 2(3):247-255.					
Ruaro. Italy. 2022	Ruaro B. Confalonieri P. Caforio G. Baratella E. Pozzan R. Tavano S. Bozzi C. Lerda S. Geri P. Biolo M et al: Chronic Thromboembolic Pulmonary Hypertension: An Observational Study. Medicina (Kaunas) 2022. 58(8).	-	-	-	1/65 (1.5%)	1/65 (1.5%)
Kvasnička. Czechia. 2024	Kvasnicka J. Jansa P. Cifkova R. Duskova D. Bobcikova P. Sevcik M. Zenahlikova Z. Kvasnicka T: The incidence of the thrombophilic SNPs rs6025. rs1799963. rs2066865. rs2289252. and rs8176719 in chronic thromboembolic pulmonary hypertension. Clin Appl Thromb Hemost 2024. 30:10760296241271369.	-	-	-	13/129 (10.1%)	12/129 (9.3%)
Ogo. Japan. 2025	Ogo T. Shinke T. Inami T. Ikeda N. Abe K. Sugiura T. Anzai A. Ito H. Taniguchi Y. Tsujino I et al: Balloon Pulmonary Angioplasty for Chronic Thromboembolic Pulmonary Hypertension: A Nationwide Prospective Multicenter Registry in Japan (J-BPA). Circ Cardiovasc Interv 2025. 18(12):e016172.	39/1202 (3.2%)	14/1202 (1.2%)	22/1202 (1.8%)	-	-
Own data. Russia. 2026		5/138 (3.6%)	5/138 (3.6%)	6/138 (4.3%)	8/138 (5.8%)	14/138 (10.1%)
All		92/3008 (3.0%)	119/3244 (3.6%)	126/3262 (4.7%)	61/759 (7.9%)	65/1179 (5.6%)

Supplementary Table S3. Summary table of antithrombin deficiency meta-analysis results.

Study	Sample size	Proportion (%)	95% CI	Weight (%)	
				Fixed	Random
Simonneau. France. 1995	47	2.128	0.0539 to 11.294	1.59	5.44
Wolf. France. 2000	46	0.000	0.000 to 7.706	1.56	5.36
Colorio. Argentina. 2001	24	4.167	0.105 to 21.120	0.83	3.34
Wong. UK. 2010	45	4.444	0.543 to 15.149	1.52	5.28
Pepke-Zaba. UK. 2011	426	0.704	0.145 to 2.044	14.14	13.79
Park. Korea. 2016	134	6.716	3.117 to 12.367	4.47	9.71
Bazmpani. Greece. 2018	27	3.704	0.0937 to 18.971	0.93	3.66
Kerr. USA. 2021	552	4.891	3.248 to 7.037	18.32	14.43
Lian. China. 2022	367	1.090	0.298 to 2.767	12.19	13.38
Ogo. Japan. 2025	1202	3.245	2.317 to 4.409	39.85	15.77
Own data. Russia. 2026	138	3.623	1.187 to 8.253	4.60	9.83
Total (fixed effects)	3008	2.961	2.386 to 3.630	100.00	100.00
Total (random effects)	3008	3.087	1.856 to 4.616	100.00	100.00

Supplementary Table S4. Test for heterogeneity of antithrombin deficiency meta-analysis results.

Q	32.4880
DF	10
Significance level	P = 0.0003
I^2 (inconsistency)	69.22%
95% CI for I^2	42.56 to 83.51

Supplementary Table S5. Publication bias of antithrombin deficiency meta-analysis results.

Egger's test	
Intercept	0.2886
95% CI	-2.0618 to 2.6389
Significance level	P = 0.7875
Begg's test	
Kendall's Tau	0.01818
Significance level	P = 0.9379

Supplementary Table S6. Summary table of protein S deficiency meta-analysis results.

Study	Sample size	Proportion (%)	95% CI	Weight (%)	
				Fixed	Random
Simonneau. France. 1995	39	0.000	0.000 to 9.025	1.23	6.33
Wolf. France. 2000	46	0.000	0.000 to 7.706	1.44	6.86
Colorio. Argentina. 2001	10	10.000	0.253 to 44.502	0.34	2.69
Wong. UK. 2010	22	0.000	0.000 to 15.437	0.71	4.59
Pepke-Zaba. UK. 2011	426	9.624	6.995 to 12.830	13.12	11.84
Park. Korea. 2016	134	4.478	1.661 to 9.491	4.15	9.91
Ogawa. Japan. 2017	308	2.273	0.919 to 4.626	9.49	11.45
Kerr. USA. 2021	552	4.710	3.100 to 6.826	16.99	12.09
Lian. China. 2022	367	5.177	3.145 to 7.967	11.31	11.68
Ogo. Japan. 2025	1202	1.165	0.638 to 1.946	36.96	12.57
Own data. Russia. 2026	138	3.623	1.187 to 8.253	4.27	9.98
Total (fixed effects)	3244	3.297	2.710 to 3.969	100.00	100.00
Total (random effects)	3244	3.562	1.824 to 5.850	100.00	100.00

Supplementary Table S7. Test for heterogeneity of protein S deficiency meta-analysis results.

Q	71.0036
DF	10
Significance level	$P < 0.0001$
I^2 (inconsistency)	85.92%
95% CI for I^2	76.59 to 91.53

Supplementary Table S8. Publication bias of protein S deficiency meta-analysis results.

Egger's test	
Intercept	0.7476
95% CI	-2.8152 to 4.3103
Significance level	$P = 0.6463$
Begg's test	
Kendall's Tau	-0.09091
Significance level	$P = 0.6971$

Supplementary Table S9. Summary table of protein C deficiency meta-analysis results.

Study	Sample size	Proportion (%)	95% CI	Weight (%)	
				Fixed	Random
Simonneau. France. 1995	39	7.692	1.615 to 20.870	1.22	5.00
Wolf. France. 2000	46	2.174	0.0550 to 11.527	1.44	5.54
Colorio. Argentina. 2001	13	7.692	0.195 to 36.030	0.43	2.25
Wong. UK. 2010	10	0.000	0.000 to 30.850	0.34	1.83
Pepke-Zaba. UK. 2011	426	8.920	6.390 to 12.038	13.04	12.39
Park. Korea. 2016	134	5.970	2.612 to 11.425	4.12	9.31
Ogawa. Japan. 2017	308	2.597	1.128 to 5.054	9.44	11.71
Bazmpani. Greece. 2018	27	7.407	0.910 to 24.290	0.86	3.90
Kerr. USA. 2021	552	4.348	2.805 to 6.400	16.89	12.84
Lian. China. 2022	367	3.542	1.899 to 5.981	11.24	12.09
Ogo. Japan. 2025	1202	1.830	1.150 to 2.758	36.74	13.75
Own data. Russia. 2026	138	4.348	1.612 to 9.223	4.25	9.41
Total (fixed effects)	3262	3.709	3.088 to 4.414	100.00	100.00
Total (random effects)	3262	4.657	3.025 to 6.618	100.00	100.00

Supplementary Table S10. Test for heterogeneity of protein C deficiency meta-analysis results.

Q	44.6913
DF	11
Significance level	$P < 0.0001$
I^2 (inconsistency)	75.39%
95% CI for I^2	56.74 to 86.00

Supplementary Table S11. Publication bias of protein C deficiency meta-analysis results.

Egger's test	
Intercept	1.4701
95% CI	-0.7386 to 3.6787
Significance level	P = 0.1689
Begg's test	
Kendall's Tau	0.06061
Significance level	P = 0.7839

Supplementary Table S12. Summary table of *F5* Leiden meta-analysis results.

Study	Sample size	Proportion (%)	95% CI	Weight (%)	
				Fixed	Random
Wolf. France. 2000	46	6.522	1.366 to 17.896	6.12	9.79
Colorio. Argentina. 2001	18	0.000	0.000 to 18.530	2.47	5.65
Suntharalingam. UK. 2008	214	7.009	3.976 to 11.297	27.99	16.01
Wong. UK. 2010	31	29.032	14.223 to 48.036	4.17	7.94
Bazmpani. Greece. 2018	27	14.815	4.189 to 33.731	3.65	7.32
Dodson. USA. 2020	91	8.791	3.872 to 16.589	11.98	12.94
Ruaro. Italy. 2022	65	1.538	0.0389 to 8.276	8.59	11.42
Kvasnička. Czechia. 2024	129	10.078	5.476 to 16.616	16.93	14.34
Own data. Russia. 2026	138	5.797	2.536 to 11.103	18.10	14.59
Total (fixed effects)	759	7.926	6.115 to 10.068	100.00	100.00
Total (random effects)	759	8.364	5.197 to 12.201	100.00	100.00

Supplementary Table S13. Test for heterogeneity of *F5* Leiden meta-analysis results.

Q	21.7243
DF	8
Significance level	P = 0.0055
I^2 (inconsistency)	63.17%
95% CI for I^2	24.33 to 82.08

Supplementary Table S14. Publication bias of *F5* Leiden meta-analysis results.

Egger's test	
Intercept	0.8707
95% CI	-2.9660 to 4.7074
Significance level	P = 0.6081
Begg's test	
Kendall's Tau	0.1111
Significance level	P = 0.6767

Supplementary Table S15. Summary table of *F2* G20210A meta-analysis results.

Study	Sample size	Proportion (%)	95% CI	Weight (%)	
				Fixed	Random
Wolf. France. 2000	40	2.500	0.0633 to 13.159	3.45	6.33
Colorio. Argentina. 2001	18	5.556	0.141 to 27.294	1.60	3.41
Suntharalingam. UK. 2008	214	4.206	1.941 to 7.833	18.08	15.74
Wong. UK. 2010	31	9.677	2.042 to 25.754	2.69	5.24
Pepke-Zaba. UK. 2011	426	3.521	1.984 to 5.741	35.91	19.05
Bazmpani. Greece. 2018	27	7.407	0.910 to 24.290	2.35	4.71
Dodson. USA. 2020	91	7.692	3.149 to 15.209	7.74	10.72
Ruaro. Italy. 2022	65	1.538	0.0389 to 8.276	5.55	8.79
Kvasnička. Czechia. 2024	129	9.302	4.900 to 15.686	10.93	12.80
Own data. Russia. 2026	138	10.145	5.658 to 16.435	11.69	13.21
Total (fixed effects)	1179	5.553	4.321 to 7.011	100.00	100.00
Total (random effects)	1179	6.179	4.200 to 8.509	100.00	100.00

Supplementary Table S16. Test for heterogeneity of *F2* G20210A meta-analysis results.

Q	17.2200
DF	9
Significance level	P = 0.0454
I^2 (inconsistency)	47.74%
95% CI for I^2	0.00 to 74.78

Supplementary Table S17. Publication bias of *F2* G20210A meta-analysis results.

Egger's test	
Intercept	1.2046
95% CI	-1.0227 to 3.4319
Significance level	P = 0.2476
Begg's test	
Kendall's Tau	0.06667
Significance level	P = 0.7884
