# Supplementary material for: Integration of hereditary thrombophilia data and polygenic risk scores into prognostic models improves differential diagnosis of CTEPH
Source: Front Cardiovasc Med. 2026 Oct 5;13:1887880. doi: 10.3389/fcvm.2026.1887880 (PMC13678304; doi:10.3389/fcvm.2026.1887880)
Supplement: Supplementary file 2 [file Datasheet4.docx]

**Supplementary Figure S1**. Article selection pipeline for inclusion in the meta-analysis.

**A**

**B**

**Supplementary Figure S2**. Details of meta-analysis for antithrombin deficiency. All regions included. **A.** Funnel plot for antithrombin deficiency meta-analysis. **B.** Forest plot for antithrombin deficiency meta-analysis.

**A**

**B**

**Supplementary Figure S3**. Details of meta-analysis for protein S deficiency. All regions included. **A.** Funnel plot for protein S deficiency meta-analysis. **B.** Forest plot for protein S deficiency meta-analysis.

**A**

.

**B**

**Supplementary Figure S4**. Details of meta-analysis for protein C deficiency. All regions included. **A.** Funnel plot for protein C deficiency meta-analysis. **B.** Forest plot for protein C deficiency meta-analysis.

**A**

**B**

**Supplementary Figure S5**. Details of meta-analysis for F5 Leiden (rs6025). Only Caucasian included. **A.** Funnel plot for F5 Leiden meta-analysis. **B.** Forest plot for F5 Leiden meta-analysis.

**A**

**B**

**Supplementary Figure S6**. Details of meta-analysis for F2 G20210A. Only Caucasian included. **A.** Funnel plot for *F2* G20210A meta-analysis. **B.** Forest plot for *F2* G20210A meta-analysis.
